# Editorial: Wearable technology: the new ornament of network physiology

**DOI:** 10.3389/fnetp.2025.1690563

**Published:** 2025-09-18

**Authors:** Ankita Srivastava, Santosh Kumar Yadav

**Affiliations:** ^1^ Daniel Baugh Institute for Functional Genomics and Computational Biology, Department of Pathology and Genomic Medicine, Thomas Jefferson University, Philadelphia, PA, United States; ^2^ Division of Hematology, Children’s Hospital of Philadelphia, Philadelphia, PA, United States

**Keywords:** wearable devices, artificial intelligence, network physiology, real-time monitoring, personalized medicine, sleep monitoring, menstrual cycle, Parkinson disease

## Introduction

The landscape of personal health monitoring is rapidly transforming, driven by advances in wearable technology. These devices, designed to be worn comfortably on the body, can capture real-time physiological data often difficult to obtain in traditional clinical settings. What began as a simple device for activity trackers for walking steps, heart rate, and blood oxygen saturation has evolved to play a crucial role in monitoring more complex physiological parameters critical for chronic disease management and preventative health.

The integration of artificial intelligence (AI) is further expanding the potential of wearable devices, enabling deeper analysis and predictive modeling of the recorded data. As AI-driven models become more refined, wearable technologies are poised to transform health monitoring, enabling early disease detection and personalized interventions. In many ways, we are at a pivotal moment in digital health, well-positioned to reimagine how healthcare is delivered, monitored, and personalized.

This editorial discusses emerging clinical applications of wearable technologies across different life stages in patient care, the key limitations, and prospects in the rapidly advancing field. These include sleep monitoring in children, menstrual cycle tracking in reproductive-aged females, and cardiovascular monitoring in Parkinson’s disease. Collectively, these studies highlight how continuous, real-time physiological data can uncover underlying subtle biological patterns and inform tailored interventions.

### Wearables in pediatric sleep monitoring

**FIGURE 1:**
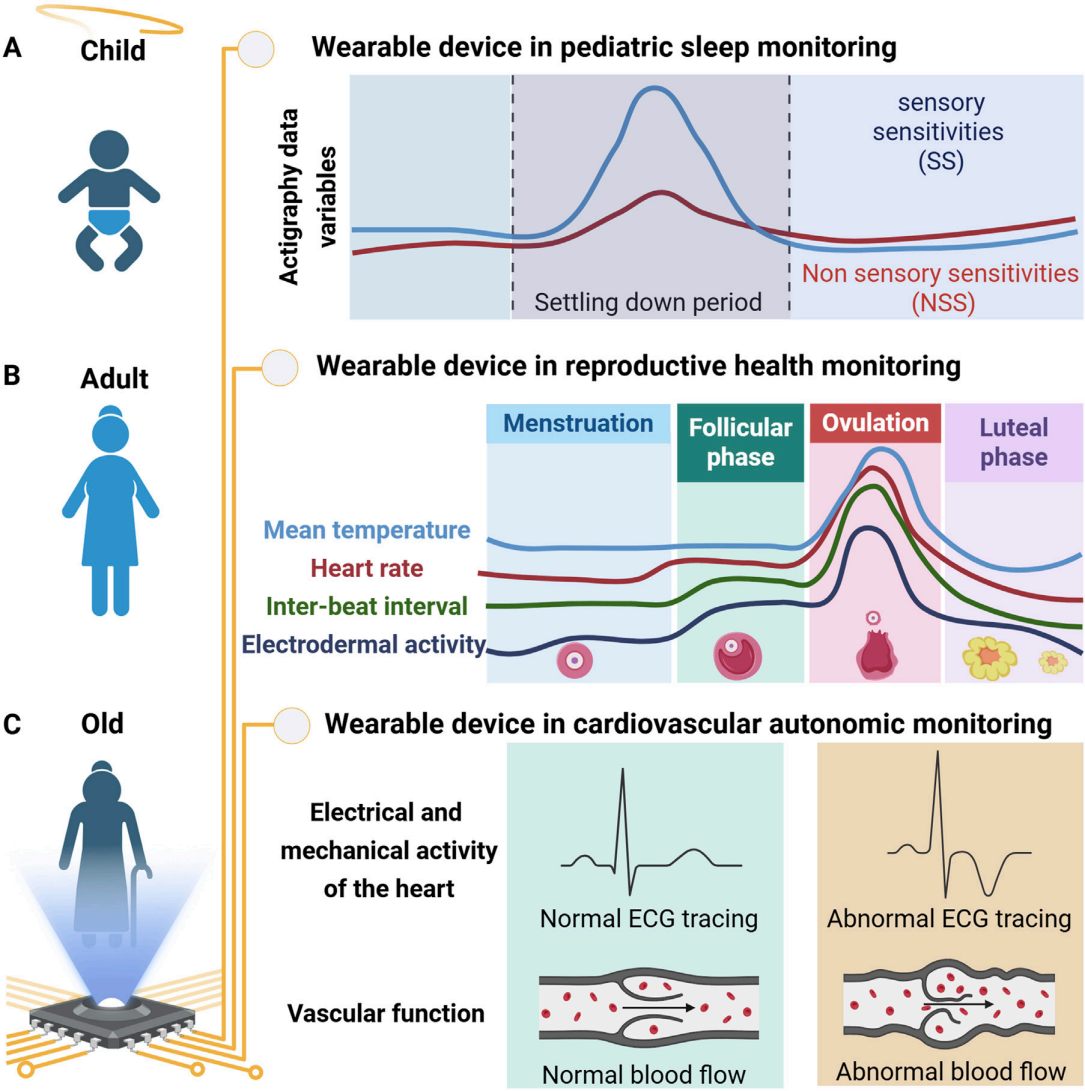
Schematic representation of physiological parameters recorded by wearable devices across different stages of life. **(A)** In pediatric sleep monitoring, wearables can distinguish between children with and without sensory sensitivities during the settling-down period using multiple parameters. **(B)** In young adult females, wearables are advancing reproductive health by tracking key reproductive cycle parameters. **(C)** In Parkinsonism, wearables capture electrical and mechanical cardiac activity alongside vascular function to monitor changes in cardiovascular autonomic (CVA) effects of levodopa.

In children, particularly those with sensory sensitivities, the settling-down period (time between the end of bedtime routine and sleep onset) can be challenging. In a recent study, Hartman and colleagues investigated how wearable devices recorded data combined with machine learning models could help characterize the differences in the settling-down period in children with and without sensory sensitivities (SS/NSS) (Kocanaogullari et al.). The study involved continuous 2-week actigraphy data (measurement of rest and activity cycles) Research Topic using the ActiGraph GT9X device worn on children with (n = 17) and without (n = 18) sensory sensitivities. Combining caregiver reports and the motor activity actigraphy data, the authors selected seven features and trained a machine learning model to distinguish between the SS and NSS groups with a high accuracy (84.1%), specificity (82.7%), and sensitivity (84.4%) (Figure 1A).

This study highlights the potential of wearables in pediatric behavioral health and opens new avenues for personalized pediatric behavior interventions. However, limitations such as small cohort size, lack of diversity in the study groups, variability in wearable device placement, and reliance on subjective caregiver reports highlight the need to use larger and diverse sample cohorts, confirm observed activity differences, and apply them in intervention studies for children with sleep difficulties. Future studies should involve a larger and more diverse sample cohort and incorporate additional physiological parameters, e.g., heart rate variability and skin temperature, to provide a more comprehensive understanding of the physiological state during the settling-down period.

### Wearables in advancing reproductive health through cycle tracking

In reproductive-aged females, wearable technologies offer novel opportunities to track the menstrual cycle and optimize menstrual health management. Nasseri’s group used a wearable device to understand female reproductive physiology and investigated menstrual cycle dynamics (Sides et al.). In this study, physiological data were recorded using wearable sensors (Empatica E4 wristband) from healthy female individuals (n = 15; 18–40 years old) across 39 cycles (31 ovulating, 8 non-ovulating). The collected data included mean temperature, heart rate, inter-beat interval, and the mean tonic component of Electrodermal Activity (EDA). The authors used circular statistics to select key features for time series forecasting using the Autoregressive Integrated Moving Average (ARIMA) algorithm. This approach accurately identified and predicted menstrual cycle phases, distinguishing between ovulating and non-ovulating cycles (Figure 1B).

The study contributes significantly to precise menstrual health management and personalised medical interventions based on distinct physiological patterns recorded by a wearable device. For instance, a reproductive-aged female undergoing medication while trying to conceive could monitor and adjust dosages in consultation with a physician according to their menstrual phase. Similarly, females with health research topic linked to a specific menstrual phase, such as catamenial epilepsy, may benefit by adjusting the anti-seizure medication dosage based on their ovulatory status. The study has limitations, including a small sample size, data variability based on the wristband positioning, and the absence of blood hormonal levels to detect ovulation. Nonetheless, accurately identifying and predicting menstrual phases and distinguishing ovulating from non-ovulating cycles holds immense promise for precise menstrual health management and individualized medical interventions.

### Wearables in cardiovascular autonomic monitoring in neurodegenerative disease

In older adults with neurodegenerative conditions, managing autonomic dysfunction is particularly challenging. Berkebile`s group evaluated the feasibility of a wearable chest patch to monitor cardiovascular autonomic (CVA) function in Parkinson’s disease (PD) patients undergoing levodopa treatment (Kehnemouyi et al.). Levodopa, the primary medication for motor symptoms in Parkinson’s disease and multiple system atrophy (MSA), can exacerbate cardiovascular autonomic (CVA) dysfunction due to its potential hypotensive effect. Monitoring CVA function is crucial, yet current approaches are limited primarily to clinical settings and symptom reporting, failing to capture its holistic nature.

In this study, a multimodal wearable chest patch was used to monitor changes in cardiovascular autonomic (CVA) dysfunction in 14 patients (11 PD, 3 MSA) under both clinical and 24-h ambulatory (at home) conditions. The device recorded electrocardiogram (ECG), seismocardiogram (SCG), and photoplethysmogram (PPG) signals during clinical “OFF” and “ON” levodopa protocols and 24-h ambulatory monitoring. The authors extracted beat-by-beat physiological markers related to CVA function using the wearable device that collected rich physiological data. The wearable patch was sensitive to CVA dynamics and provided preliminary insights into levodopa’s effects on cardiac contractility and autonomic regulation, particularly in patients with orthostatic hypotension (Figure 1C). The study provides a promising framework for exploring levodopa’s acute CVA effects and highlights the potential of wearable-derived physiomarkers to inform personalized care strategies for people with Parkinsonism. The study is limited by a small number of participants combined with a heterogeneous cohort, including PD and MSA patients, constraining the statistical modeling and inferences based on the disease condition. Furthermore, the 24-h ambulatory monitoring was uncontrolled and influenced by factors like eating, mental state, and concurrent medications, introducing variability in levodopa response analysis. The study lays the foundation for further exploring wearable-derived physiological markers for personalized care in Parkinson’s disease. Future studies should aim to include a larger, more diverse cohort. Furthermore, integrating physical activity and posture could provide a more comprehensive CVA monitoring solution.

## Discussion

Wearable technologies are reshaping the landscape of personal health monitoring, enabling continuous, real-time physiological data Research Topic outside traditional clinical assessments. The growing role of wearable devices across diverse health domains presents exciting opportunities for precision health monitoring and intervention. The studies highlighted in this commentary demonstrate how the rich data obtained from wearable devices, paired with advanced statistical analysis and machine learning, can yield clinically relevant insights. From identifying sleep behavior patterns in children with sensory sensitivities to improve sleep routines, to monitoring menstrual physiology enabling better management of menstrual health, and informing therapeutic strategies in Parkinson’s disease, wearable technologies are unlocking potential opportunities to better understand physiological dynamics and tailor interventions to individual patient needs. Collectively, these studies highlight several overarching themes that enable a deeper understanding of the interplay within our physiological networks. First, integrating advanced data analytics, including AI models and machine learning, is essential to extracting meaningful and actionable insights from wearable device-generated rich data. Second, shifting from episodic, clinic-based measurements to continuous, real-world monitoring empowers patients and clinicians with a more holistic and dynamic understanding of health and disease.

However, several challenges remain, including a small sample size that limits generalizability, variability in sensor placement that can affect data quality, subjective inputs, such as caregiver reports or the absence of biochemical confirmation to validate physiological data, all of which constrain interpretability. Future studies should prioritize larger and more diverse cohorts, standardized sensor protocols, and integrating multimodal physiological and biochemical markers to validate wearable-derived data. Such efforts are pursued in interdisciplinary collaboration between clinicians, engineers, data scientists, and patients. Moreover, ethical considerations, particularly around data privacy, user engagement, and equitable access, must also be addressed to ensure these technologies benefit all populations. As wearable devices continue to evolve in accuracy, accessibility, and analytical power, they hold the promise to transform our understanding of human physiology and advance personalized health across the human lifespan. The “new ornament of network physiology” is not merely a fashion statement but a fundamental shift in how we perceive, measure, and manage human health.

